# Effect of Insertion and Deletion in the Meq Protein Encoded by Highly Oncogenic Marek’s Disease Virus on Transactivation Activity and Virulence

**DOI:** 10.3390/v14020382

**Published:** 2022-02-14

**Authors:** Jumpei Sato, Shiro Murata, Zhiyuan Yang, Benedikt B. Kaufer, Sotaro Fujisawa, Hikari Seo, Naoya Maekawa, Tomohiro Okagawa, Satoru Konnai, Nikolaus Osterrieder, Mark S. Parcells, Kazuhiko Ohashi

**Affiliations:** 1 Department of Disease Control, Faculty of Veterinary Medicine, Hokkaido University, Sapporo 060-0818, Japan; s16110010@st.obihiro.ac.jp (J.S.); b20203050402@cau.edu.cn (Z.Y.); s.fujisawa@vetmed.hokudai.ac.jp (S.F.); seo-fuwago@eis.hokudai.ac.jp (H.S.); konnai@vetmed.hokudai.ac.jp (S.K.); okazu@vetmed.hokudai.ac.jp (K.O.); 2 Department of Advanced Pharmaceutics, Faculty of Veterinary Medicine, Hokkaido University, Sapporo 060-0818, Japan; maekawa@vetmed.hokudai.ac.jp (N.M.); okagawa@vetmed.hokudai.ac.jp (T.O.); 3 Institute of Animal Husbandry and Veterinary Medicine, Beijing Academy of Agriculture and Forestry Sciences, Beijing 100097, China; 4 Institut für Virologie, Freie Universität Berlin, 14163 Berlin, Germany; benedikt.kaufer@fu-berlin.de (B.B.K.); no.34@fu-berlin.de (N.O.); 5 Department of Infectious Diseases and Public Health, City University of Hong Kong, Kowloon Tong, Hong Kong; 6 Department of Animal and Food Sciences, University of Delaware, Newark, DE 19716, USA; parcells@udel.edu

**Keywords:** Marek’s disease virus, Marek’s disease, Meq, CVI988, tumorigenesis, pathogenicity, transactivation activity

## Abstract

Marek’s disease virus (MDV) causes malignant lymphoma in chickens (Marek’s disease, MD). Although MD is currently controlled by vaccination, MDV strains have continuously increased in virulence over the recent decades. Polymorphisms in Meq, an MDV-encoded oncoprotein that serves as a transcription factor, have been associated with the enhanced virulence of the virus. In addition, insertions and deletions in Meq have been observed in MDV strains of higher virulence, but their contribution to said virulence remains elusive. In this study, we investigated the contribution of an insertion (L-Meq) and a deletion in the Meq gene (S-Meq) to its functions and MDV pathogenicity. Reporter assays revealed that both insertion and deletion enhanced the transactivation potential of Meq. Additionally, we generated RB-1B-based recombinant MDVs (rMDVs) encoding each Meq isoform and analyzed their pathogenic potential. rMDV encoding L-Meq indueced the highest mortality and tumor incidence in infected animals, whereas the rMDV encoding S-Meq exhibited the lowest pathogenicity. Thus, insertion enhanced the transactivation activity of Meq and MDV pathogenicity, whereas deletion reduced pathogenicity despite having increased transactivation activity. These data suggest that other functions of Meq affect MDV virulence. These data improve our understanding of the mechanisms underlying the evolution of MDV virulence.

## 1. Introduction

Marek’s disease virus (MDV) is an alphaherpesvirus that belongs to the Herpesviridae family (subfamily: *Alphaherpesvirinae*, genus: *Mardivirus*, species: *Gallid alphaherpesvirus 2*). MDV causes Marek’s disease (MD), which is characterized by neurological disorders, malignant lymphomas, and immunosuppression in infected chickens. Although MD has previously caused severe economic losses in the poultry industry, the disease is currently controlled by administering attenuated and/or non-pathogenic strains as vaccines [1]. *Meleagrid alphaherpesvirus 1* (turkey herpesvirus, HVT) was initially introduced to the poultry industry, followed by bivalent vaccines consisting of HVT and a strain of non-oncogenic *Gallid alphaherpesvirus 3*, and an attenuated *Gallid alphaherpesvirus 2* strain, Rispens CVI988, was introduced and is widely used as a gold standard vaccine against MD [2]. Introduction of the vaccines minimized the economic losses caused by virulent MDVs; however, the virulence of MDV field strains has continuously increased. Sporadic occurrences of MD are still reported even in vaccinated flocks in some countries [3,4,5,6,7,8], and the costs to the poultry industry are estimated at approximately USD 2 billion annually [9]. The emergence of MDV strains with increased virulence is considered to be a result of the selection of field strains adapted to vaccine-induced immune pressure [10,11,12]. Therefore, MDV strains with higher virulence may cause future outbreaks [13]. 

The *meq* oncogene encodes a 339-amino-acid protein that is expressed during the lytic and latent phases of infection and is essential for transformation of chicken T-cells [14]. Meq consists of a basic region (BR) and a leucine zipper (ZIP), similar to those of the oncoproteins Jun and Fos, at the N-terminus, and a transactivation domain characterized by proline-rich repeats (PRRs) at the C-terminal [14]. Meq can dimerize with itself, c-Jun, JunB, and Fos through its ZIP domain [15]. Microarray analysis identified that the same genes induced by v-Jun transformation, including *JTAP1*, *JAC*, and *HB-EGF*, were also upregulated by the expression of Meq in transformed DF-1 cells [16]. Additionally, Meq upregulated the expression of Bcl-2 and Ski and downregulated the expression of DAP5 and Fas, consistent with its anti-apoptotic properties [16,17]. Furthermore, *meq*-deleted recombinant MDV (rMDV) failed to induce lymphomas in infected chickens, indicating that Meq plays an essential role in the transformation induced by virulent MDV [18]. The distinct diversity in Meq proteins of MDV strains with varying virulence has been highlighted as a factor involved in the increased virulence of MDV [19]. Among them, the polymorphisms in the BR and PRRs in the transactivation domain have been associated with virulence-level, and several amino acid substitutions in these regions affect the transactivation activities of Meq proteins [20,21,22]. Moreover, in vivo studies using rMDVs generated based on the very virulent strain RB-1B strain revealed that rMDVs encoding Meq from highly virulent MDV strains showed higher virulence than those from low virulent strains [23]. Thus, polymorphisms in the *meq* gene appear to be directly involved in the increased virulence of MDV field strains.

In addition to the polymorphisms in Meq proteins, insertion or deletion in the transactivation domain of Meq have been detected in several MDV strains [8,20,24,25,26,27,28]. A long isoform of Meq containing an insertion (L-Meq) is characterized by a 59/60-amino acid insertion in the transactivation domain [24,27,29], and its insertion causes an increase in the number of PRRs. L-Meq was first identified in CVI988 [29] and has subsequently been detected in low-virulence strains isolated in the USA [19]. Therefore, it was initially believed that the L-Meq isoform contributes to decreased virulence of MDVs encoding this form. However, it was clarified that virulent MDV strains circulating in Australia encode an L-Meq, which also contains polymorphisms in PRRs [24]. Furthermore, recent in vivo experiments using RB-1B-based rMDVs harboring L-Meq or Meq encoded by CVI988, rMDV encoding CVI988-L-Meq exhibited markedly higher pathogenicity and tumorigenicity than did CVI988 [30]. Surprisingly, the rMDV encoding CVI988-L-Meq showed similar or even greater virulence than the rMDV encoding RB-1B-Meq, although RB-1B is an MDV strain with high virulence. Thus, amino acid insertions into the transactivation domain appear to contribute to enhanced virulence.

Since the 2010s, MDV strains encoding the short isoform of Meq (S-Meq) have been detected in some countries [20,25,28,31]. S-Meq contains a 41-amino acid deletion in the transactivation domain, resulting in a decrease in the number of PRRs. In addition, an in vitro study revealed that S-Meq had higher transactivation activity than Meq, suggesting that deletion in the transactivation domain enhances the protein functions of Meq [20]. However, MDV strains encoding S-Meq were observed in chickens without clinical signs in Italy [25] and in diseased but unvaccinated chickens in Japan [20]. Therefore, the contribution of S-Meq to MDV pathogenicity remains unclear. However, because deletion in the transactivation domain was observed in recent field strains and could enhance the transactivation activity of Meq, this deletion may be involved in the evolution towards a greater virulence of these strains [20]. 

In this study, we systematically investigated the contribution of a common insertion and deletion in the transactivation domain of Meq and evaluated these for their effects on transactivation and MDV pathogenicity. We analyzed the transactivation activities of Meq, L-Meq, and S-Meq, the background sequences of which were identical to those of RB-1B-Meq. In addition, we generated RB-1B-based rMDVs encoding each Meq isoform by replacing the *meq* gene of the RB-1B genome cloned as a bacterial artificial chromosome (BAC) with the L-*meq*/S-*meq* gene that was introduced, and animal experiments were conducted to examine whether insertion/deletion affected MDV pathogenicity.

## 2. Materials and Methods

### 2.1. Ethics Statement

All animal experiments were approved by the Institutional Animal Care and Use Committee of Hokkaido University (approval number: 19-0081). All experiments were performed in accordance with the relevant guidelines and regulations of the Faculty of Veterinary Medicine of Hokkaido University, which is fully accredited by the Association for Assessment and Accreditation of Laboratory Animal Care International. 

### 2.2. Cells and Virus

Chicken embryo fibroblasts (CEFs) were prepared from 10-day-old fertilized eggs (Iwamura Hatchery Co., Ltd., Shibata, Japan) as described previously [32]. CEFs were cultured in Eagle’s minimal essential medium (Nissui Pharmaceutical Co., Ltd., Tokyo, Japan) supplemented with 10% bovine calf serum (Sigma-Aldrich, St. Louis, MO, USA), 10% tryptose phosphate broth (Difco Laboratories, Detroit, MI, USA), 0.03% L-glutamine, 100 U/mL penicillin, 100 μg/mL streptomycin, and 0.1% NaHCO_3_. DF-1 cells, a chicken fibroblast cell line, were cultured with 0.5 mL of Dulbecco’s modified Eagle’s medium (FUJIFILM Wako Pure Chemical Corporation, Osaka, Japan), containing 10% fetal bovine serum, and incubated at 39 °C under 5% CO_2_. Viruses were reconstituted by transfecting BAC DNA into CEFs as described previously [33]. Viruses were propagated on CEFs for seven passages, and virus stocks were frozen in Cell Banker 1 (Nippon Zenyaku Kogyo Co., Ltd., Fukushima, Japan) and titrated on CEFs using plaque assays as described previously [34,35].

### 2.3. Plasmids

The expression plasmids for S-Meq and L-Meq were constructed, and some mutations were introduced by site-directed mutagenesis, according to a previous report [22]. The open reading frames (ORFs) of the S-*meq* and L-*meq* genes derived from the MDV strains Kgw-c2, an MDV strain isolated from unvaccinated chickens with MD symptoms in Japan in the 2010s (accession number: LC385874), and CVI988, an attenuated vaccine strain (accession number: AF493555) were amplified and cloned into the pCI-neo vector (Promega, Madison, WI, USA). The pathotype of Kgw-c2 has not been determined. To match the amino acid sequences with those of Meq from the very virulent RB-1B strain, aside from deletion or insertion in the transactivation domain, we introduced some mutations in the S-*meq* and L-*meq* genes, as shown in Table 1, and their S-Meq and L-Meq were designated as S-Meq (RB-1B) and L-Meq (RB-1B), respectively. In addition, we constructed an expression plasmid for Meq from MDV strain RB-1B (accession number: HM488349.1). For the assay to measure the transactivation activity, a c-Jun expression plasmid was constructed [22], and a reporter plasmid was prepared by inserting the Meq promoter region upstream of the firefly luciferase-coding region in the pGL3-Basic vector (Promega) [22]; a pRL-TK Renilla luciferase plasmid (Promega) was used as the control plasmid.

### 2.4. Dual-Luciferase Reporter Assay

First, DF-1 cells were seeded in 24-well plates at 2.0 × 10^5^ cells per well and incubated for 24 h. The cells in each well were transfected with 300 ng of expression plasmids, Meq, S-Meq (wild-type), S-Meq (RB-1B), L-Meq (wild-type), or L-Meq (RB-1B); 200 ng of the c-Jun expression plasmid; 500 ng of the reporter plasmid; and 5 ng of control pRL-TK using Lipofectamine 2000 (Thermo Fisher Scientific, Waltham, MA, USA) according to the manufacturer’s instructions. The transfected cells were lysed 24 h after transfection using 1 × Passive Lysis Buffer (Promega). Luciferase activity was measured using the Dual-Luciferase Reporter Assay System (Promega) and a Luminescencer-JNR AB-2100 (Atto Corp., Tokyo, Japan). The luminescence intensity of firefly luciferase was normalized to that of *Renilla* luciferase, and the results are indicated relative to the value of the luciferase activity in cells transfected with the pCI-neo vector.

### 2.5. Generation of Recombinant Viruses

To generate recombinant viruses encoding S-Meq (RB-1B), Meq, or L-Meq (RB-1B), we used a BAC clone of the very virulent RB-1B strain that lacks most of the internal repeat long (IRL) region as described previously [35]. The deleted IRL region is rapidly restored during virus reconstitution in the cell culture, resulting in a recombinant virus that harbors the respective *meq* gene in both repeat regions [35]. Therefore, we replaced the *meq* gene in the terminal repeat long (TRL) with the RB-1B-*meq*, S-*meq* (RB-1B), or L-*meq* (RB-1B) gene by two-step Red-mediated mutagenesis as previously described [36,37]. To screen for clones in which each *meq*-isoform was accurately inserted, the obtained plasmids encoding each rMDV genome were digested with BamHI-HF (New England Biolabs Japan Inc., Tokyo, Japan) overnight and subjected to electrophoresis on a 0.8% agarose gel. The insertion of each *meq*-isoform was further confirmed by polymerase chain reaction (PCR) and DNA sequencing, as previously reported [35]. The BAC-based infectious clones were transfected into CEFs using a CalPhos Mammalian Transfection Kit (Takara Bio Inc., Kyoto, Japan) according to the manufacturer’s instructions. The pCAGGS-Cre plasmid (Gene Bridges GmbH, Heidelberg, Germany) was co-transfected to remove the BAC sequence from the virus genome. The reconstituted recombinant viruses were referred to as vRB-1B_Meq, vRB-1B_S-Meq, and vRB-1B_L-Meq, respectively, and all viruses were passaged on CEFs. As the IRL region is rapidly restored during viral reconstitution, the restoration of the IRL, in addition to the deletion of the BAC sequence, in each virus was confirmed by PCR [35]. Each virus was titrated using a plaque assay and stored as described previously [34,35].

### 2.6. In Vitro Replication of the Recombinant Viruses

CEFs were seeded in six-well plates and infected with 50 plaque-forming units (PFU) of recombinant viruses after reaching confluence. The infected cells were collected daily for 5 days. The viral loads in the infected cells were analyzed by quantitative PCR (qPCR) to assess the replication and spread of recombinant viruses in vitro.

### 2.7. Confirmation of Meq Expression Levels by RT-PCR

A reverse transcription (RT)-PCR assay was used to investigate the mRNA expression of each *meq* isoform. In brief, we first seeded CEFs in 24-well plates at 2.0 × 10^5^ cells per well, and after 24 h incubation, the CEFs were infected with 50 PFU of recombinant viruses. At 6 days post-infection (dpi), the infected CEFs were lysed with TRI reagent (Molecular Research Center, Inc., Cincinnati, OH, USA) and the total RNA was extracted according to the manufacturer’s instructions. After the total RNA was treated with DNase I (Promega), cDNA was synthesized using PrimeScript Reverse Transcriptase (Takara Bio Inc.). The expression of each *meq* isoform in the cDNA samples was detected by PCR using TaKaRa Ex Taq (Takara Bio Inc.). The primer sets used are listed in Table 2.

### 2.8. In Vivo Characterization of Recombinant Viruses

#### 2.8.1. Experimental Chickens

Fertilized eggs (Iwamura Hatchery Co., Ltd.) from commercial white leghorn chickens were hatched in the authors’ laboratory and the chicks were raised in isolators. To analyze the recombinant viruses, we performed animal experiments as follows:

#### 2.8.2. 1st Animal Experiment

A total of 98 1-day-old chicks (Iwamura Hatchery Co., Ltd.) were randomly divided into four groups and housed separately in different isolators. The chickens were inoculated via the intraabdominal route with 5000 PFU of vRB-1B_Meq (*n* = 28), vRB-1B_S-Meq (*n* = 27), vRB-1B_L-Meq (*n* = 27), or PBS (*n* = 16) as a negative control.

- In vivo kinetics of recombinant viruses

Four chickens per group were euthanized at 7, 14, 28, and 35 dpi, and the blood, spleens, and feather tips were collected. Peripheral blood mononuclear cells (PBMCs) were isolated by density gradient centrifugation using Percoll solution (GE Healthcare, Chicago, IL, USA). DNA was extracted from the PBMCs, spleens, and feather tips, and the viral loads were analyzed for each sample.

- Pathogenicity of recombinant viruses in unvaccinated chickens

The pathogenicity of the recombinant viruses in infected chickens, vRB-1B_Meq (*n* = 12), vRB-1B_S-Meq (*n* = 11), and vRB-1B_L-Meq (*n* = 12) were compared by monitoring the survival rate. After inoculating the chickens with recombinant viruses, we monitored them for clinical signs of MD daily for 8 weeks, euthanized the infected chickens that showed neurological symptoms and dysphagia during the experimental period, and examined the gross tumor lesions in the internal organs. In addition, we examined the tumor incidence in infected chickens without clinical signs at 56 dpi. We collected the blood, spleens, feather tips, and tumors from euthanized chickens and analyzed the viral loads in each tissue type. 

#### 2.8.3. 2nd Animal Experiment: Pathogenicity of Recombinant Viruses in Vaccinated Chickens

A total of 29 1-day-old chicks (Iwamura Hatchery Co., Ltd.) were randomly divided into three groups and housed separately. The chickens were subcutaneously inoculated with 2000 PFU of HVT vaccine (strain FC 126; Kyoritsu Seiyaku, Tokyo, Japan). At 3 days post-vaccination, the chickens were superinfected via the intraabdominal route with 5000 PFU of vRB-1B_Meq (*n* = 9), vRB-1B_S-Meq (*n* = 8), or vRB-1B_L-Meq (*n* = 12), and the survival rate and tumor incidence were monitored for 8 weeks. We collected blood, spleens, feather tips, and tumors from euthanized chickens and analyzed the viral loads in each tissue type. To monitor the viral loads, we also collected blood from the wing veins of four chickens per group at 7, 14, 28, 35, and 49 dpi and monitored the viral loads in the samples from the whole blood of infected chickens.

### 2.9. DNA Extraction

Total cellular DNA was extracted from the whole blood samples of infected chickens using a DNeasy blood and tissue kit (Qiagen, Düsseldorf, Germany) according to the manufacturer’s instructions. Total cellular DNA was extracted from the feather tips as previously described [38,39]. Briefly, the two feather tips were cut and immersed overnight at 55 °C in 1 mL of lysis buffer (0.5% sodium dodecyl sulfate, 0.1 M NaCl, 10 mM Tris pH 8.0, 1 mM ethylenediaminetetraacetic acid) containing proteinase K at a final concentration of 200 mg/mL. Total cellular DNA was extracted using phenol-chloroform-isoamyl alcohol (25:24:1), precipitated with ethanol, and treated with RNase A. The total cellular DNA was extracted from the infected CEFs, PBMCs, spleens, and tumor lesions using SepaGene (Sekisui Medical Co., Ltd., Tokyo, Japan) according to the manufacturer’s instructions. All samples were subjected to qPCR analysis to determine the viral load. 

### 2.10. qPCR

The viral loads in CEFs and chickens infected with the recombinant viruses and exposed to the HVT vaccine were determined by qPCR with primers specific to the *infected cell protein 4* (*ICP4*) gene of MDV and the *HVT070* gene of HVT, respectively. qPCR was performed using TB Green Premix DimerEraser (Takara Bio Inc.) and LightCycler 480 System II (Roche Diagnostics, Mannheim, Germany). The chicken *inducible nitric oxide synthase* (*iNOS*) gene was amplified as a reference gene. The viral loads are indicated as ratios between each target and the *iNOS* gene. The primers used for the qPCR analyses are shown in Table 2. 

### 2.11. Statistical Analyses

Statistical analyses were performed using R Statistical Software (version 4.0.3; R Foundation for Statistical Computing, Vienna, Austria). The multi-step growth kinetics were analyzed using the Kruskal–Wallis test. Kaplan–Meier survival curves were generated to analyze the survival rate in infected chickens, and a log-rank test (Mantel–Cox test) was conducted. Tumor incidence was analyzed using Fisher’s exact test. The transactivation activities were analyzed using Tukey’s multiple comparison test. Statistical significance was set at *p* < 0.05.

## 3. Results

### 3.1. Transactivation Activity of Meq Isoforms

**Figure 1:**
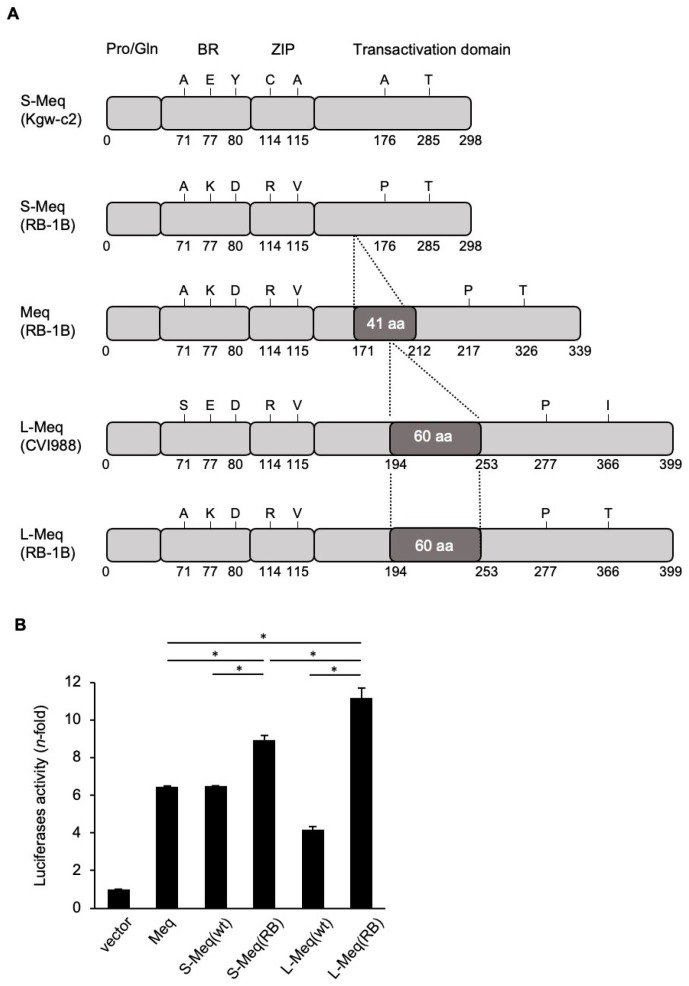
Analysis of the transactivation activity of the three Meq isoforms. (**A**) Schematic representation of the different Meq isoforms. The structures of short-Meq containing the deletion (S-Meq) from Kgw-c2, a Marek’s disease virus (MDV) strain, Meq from RB-1B, and long-Meq containing the insertion (L-Meq) from CVI988, and S-Meq (RB-1B) and L-Meq (RB-1B) whose sequences were matched with that of RB-1B-Meq, except for the deletion or insertion in the transactivation domain, are indicated. Meq comprises a proline/glutamine (Pro/Gln) rich domain followed by the basic region (BR) and leucine zipper (ZIP) at the N-terminal region, and the transactivation domain at the C-terminal region. The Meq isoforms include mutations in the BR, ZIP, and transactivation domain. S-Meq contains a 41 amino acid deletion in the transactivation domain and L-Meq is characterized by a 60-amino acid insertion in the transactivation domain. (**B**) Transactivation activity of the Meq isoforms. The transactivation activity of RB-1B-Meq, wild-type S-Meq (Kgw-c2), wild-type L-Meq (CVI988), S-Meq (RB-1B), and L-Meq (RB-1B) was compared on the Meq promoter-driven luciferase activities. DF-1 cells in each well were transfected with 300 ng of expression plasmids, Meq, S-Meq (wild-type), S-Meq (RB-1B), L-Meq (wild-type), or L-Meq (RB-1B); 200 ng of the c-Jun expression plasmid; 500 ng of the reporter plasmid; and 5 ng of control pRL-TK. Luciferase activities were analyzed 24 h post-transfection. Firefly luciferase activity is expressed relative to the mean basal activity in the presence of pCI-neo after normalization to Renilla luciferase activity. Error bars indicate standard deviations. * *p* < 0.01.

To determine whether the insertion/deletion in the transactivation domain of Meq affects protein function, we analyzed the transactivation activities of S-Meq from Kgw-c2, an MDV strain isolated in Japan in the 2010s, the Meq from RB-1B (parent), and the L-Meq from CVI988. Because there are some polymorphisms among RB-1B-*meq*, Kgw-c2-S-*meq*, and CVI988-L-*meq* (Figure 1A), we introduced some mutations in wild-type S-*meq* and L-*meq* genes from Kgw-c2 and CVI988, respectively, and manipulated their sequences to the same sequence as RB-1B-*meq*, aside from the insertion or deletion in the transactivation domain (Figure 1A). S-Meq (wild type) demonstrated similar transactivation activity to that of RB-1B-Meq, whereas the transactivation activity of L-Meq (wild type) was significantly lower than that of RB-1B-Meq and S-Meq (wild type) (Figure 1B). The mutation-introduced S-Meq, L-Meq, S-Meq (RB-1B), and L-Meq (RB-1B) showed higher transactivation activities than wild-type S-Meq and L-Meq (Figure 1B), suggesting that these amino acid substitutions were responsible for higher transactivation activity. Moreover, the transactivation activities of S-Meq (RB-1B) and L-Meq (RB-1B) were higher than that of the wild-type RB-1B Meq. Interestingly, the L-Meq (RB-1B) exhibited the highest activity among the mutated L-Meq and S-Meq, and RB-1B-Meq isoforms (Figure 1B). These data indicate that both insertion and deletion in the transactivation domain have the potential to enhance the transactivation activity of Meq.

### 3.2. Generation of Recombinant Viruses

**Figure 2:**
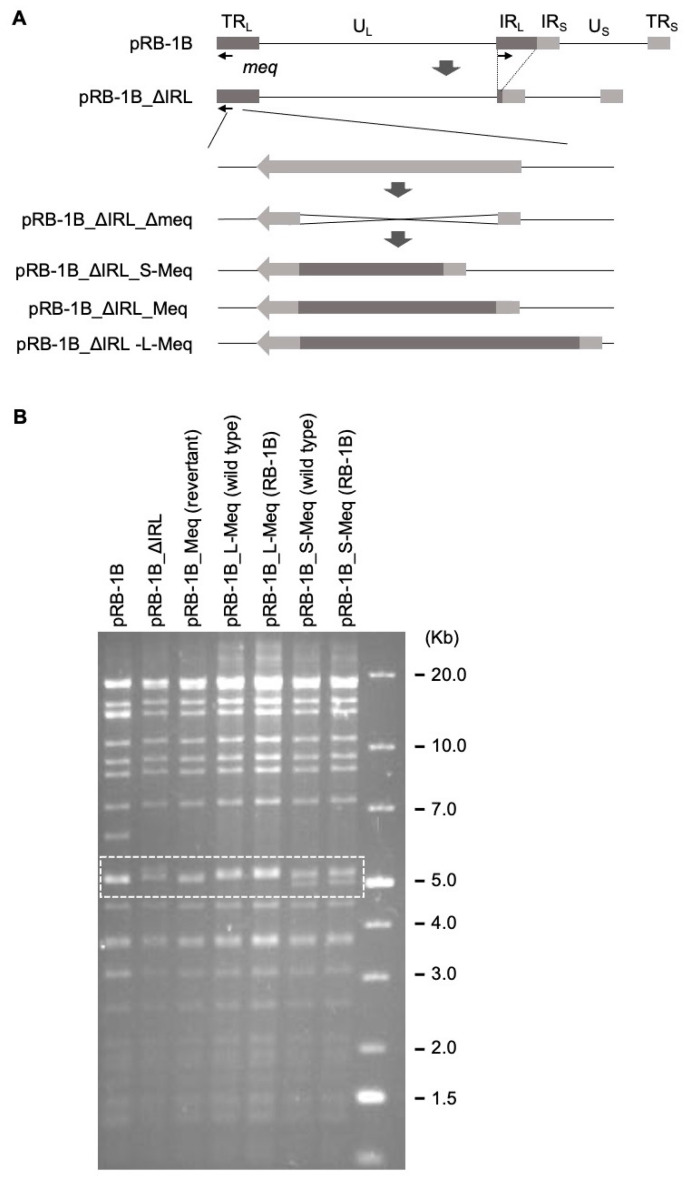
Construction of recombinant Marek’s disease viruses. (**A**) Schematic diagrams of the constructs cloned the RB-1B genome used in this study. In the RB-1B genome cloned as the bacterial artificial chromosome (BAC) plasmid (pRB-1B), most of the internal repeat long (IRL) regions were deleted, designated as pRB-1B_ΔIRL, and used for mutagenesis. The *meq* gene in terminal repeat long (TRL) was replaced with the RB-1B-*meq*, short-Meq containing the deletion (S-*meq*) (RB-1B), or long-Meq containing the insertion (L-*meq*) (RB-1B) genes by two-step red-mediated mutagenesis. (**B**) Restriction fragment length polymorphism analysis of the BAC plasmids obtained by mutagenesis. The BAC plasmids were digested with BamHI to determine the accurate insertion of each *meq*-isoform into the RB-1B genome. A dashed box indicates the variation of the recombinant Marek’s disease virus genomes with each *meq*-isoform.

To investigate whether insertion/deletion affects MDV pathogenicity, we generated RB-1B-based rMDVs encoding each Meq isoform, as previously reported [30]. We inserted the S-*meq* (RB-1B), RB-1B *meq*, and L-*meq* (RB-1B) genes into the IRL-deleted RB-1B genome cloned as a BAC plasmid by replacing the native *meq* gene in the TRL with them (Figure 2A). The resulting BAC plasmids were screened using restriction fragment length polymorphism analysis (Figure 2B and Figure S1), and the insertion of each *meq*-isoform was confirmed by polymerase chain reaction (PCR) and DNA sequencing (data not shown). The BAC-based infectious clones were transfected into chicken embryo fibroblasts (CEFs), and the restoration of the IRL in the reconstituted viruses, termed vRB-1B_S-Meq, vRB-1B_Meq, and vRB-1B_L-Meq, was confirmed by PCR (data not shown).

### 3.3. Characterization of rMDVs In Vitro

**Figure 3:**
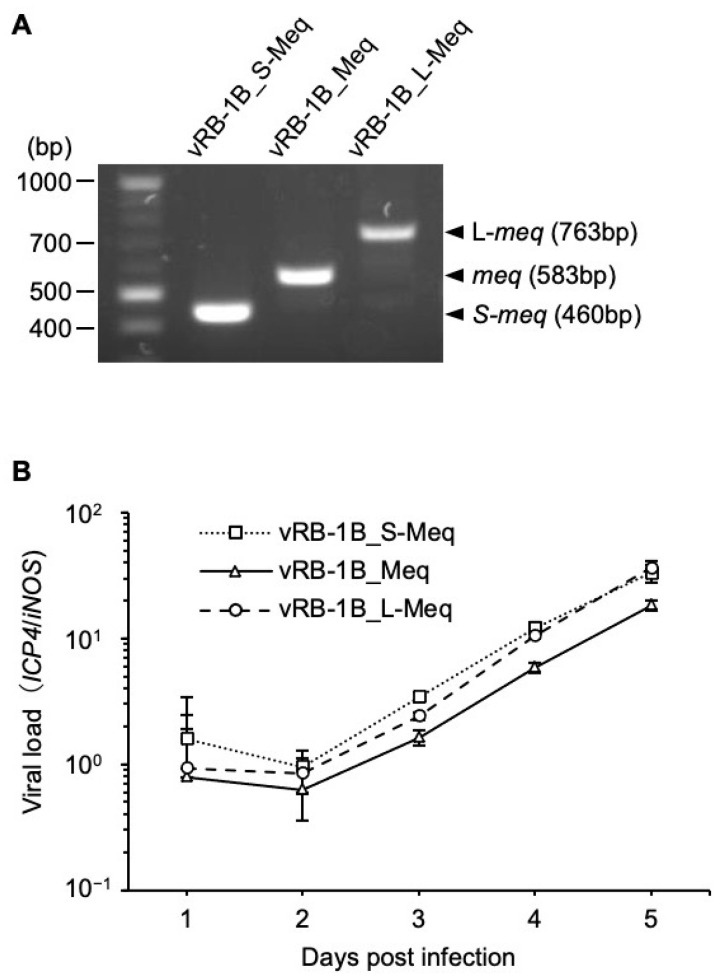
Expression analysis and replication of recombinant MDVs in cell culture. (**A**) mRNA expression of each *meq*-isoform in chicken embryo fibroblasts (CEFs) infected with vRB-1B_S-Meq, vRB-1B_Meq, and vRB-1B_L-Meq was confirmed by reverse transcription-polymerase chain reaction (PCR). (**B**) CEFs were infected with 50 plaque-forming units of recombinant Marek’s disease viruses. The infected cells were collected daily for 6 days. The viral loads in the infected cells were analyzed by quantitative PCR. The growth kinetics among the groups were analyzed using the Kruskal–Wallis test.

To determine if insertion and deletion in the transactivation domain affect virus replication in vitro, we analyzed the expression of each *meq*-isoform and viral loads in CEFs infected with vRB-1B_S-Meq, vRB-1B_Meq, and vRB-1B_L-Meq. We confirmed the mRNA expression of each *meq*-isoform in infected CEFs by RT-PCR (Figure 3A and Figure S2), and no significant difference in the growth kinetics among the rMDVs in vitro was observed (Figure 3B). These results suggest that insertion and deletion in the transactivation domain do not affect virus replication of the recombinant viruses in cell culture.

### 3.4. Replication of rMDVs In Vivo

**Figure 4:**
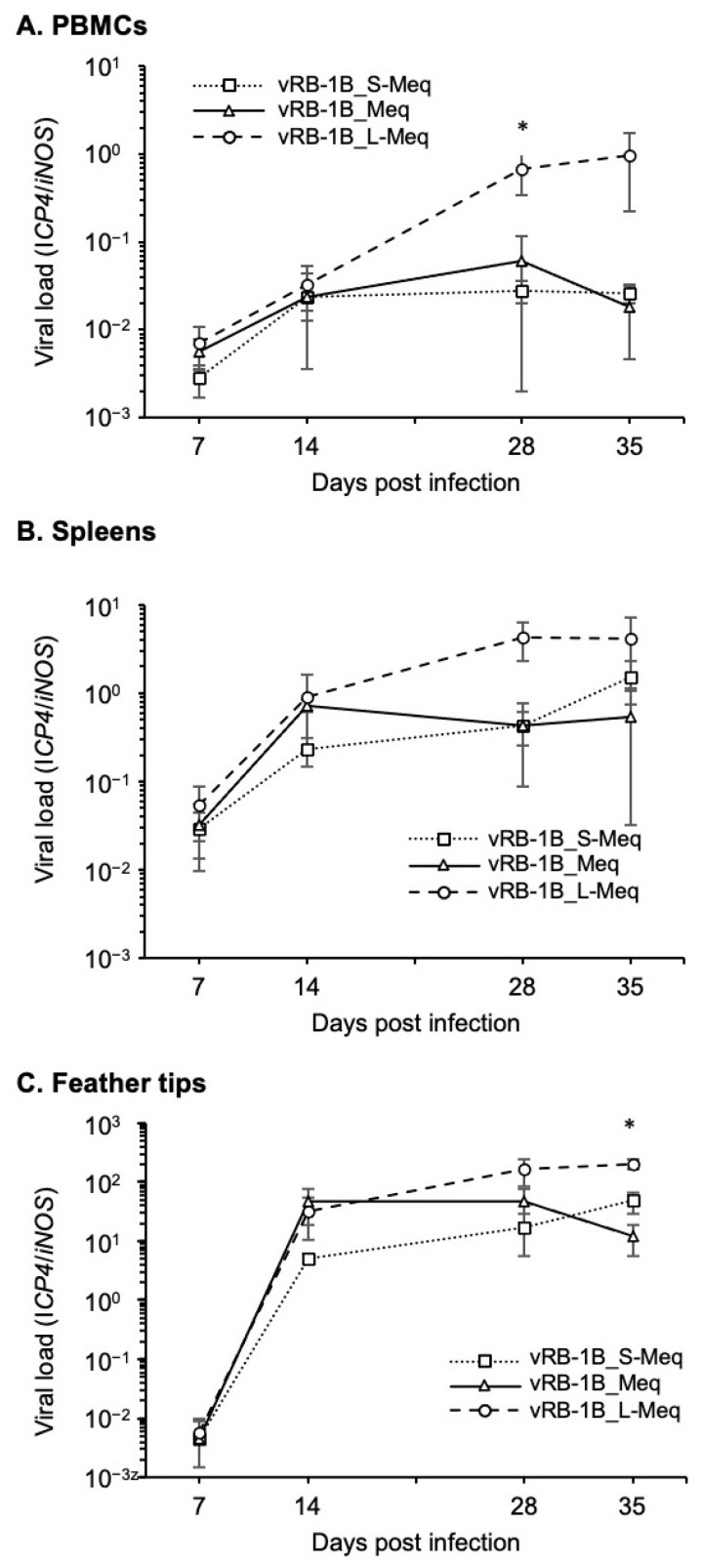
Replication of recombinant MDVs in vivo. The viral loads in the (**A**) peripheral blood mononuclear cells, (**B**) spleens, and (**C**) feather tips from chickens infected with vRB-1B_S-Meq, vRB-1B_Meq, and vRB-1B_L-Meq were determined by quantitative polymerase chain reaction. Asterisks indicate significant differences (* *p* < 0.01; Kruskal–Wallis test).

We then assessed viral replication in vivo. Day-old chicks were inoculated with 5000 PFU of each rMDV via the intraabdominal route, and the viral loads were analyzed in the PBMCs, spleens, and feather tips from four chickens per group at 7, 14, 28, and 35 dpi. Although all of the rMDVs efficiently replicated in infected birds, vRB-1B_L-Meq showed higher viral loads at 28 and 35 dpi than those of vRB-1B_S-Meq and vRB-1B_Meq (Figure 4). 

Remarkably, the viral loads in PBMCs at 28 dpi and in feather tips at 35 dpi from chickens infected with vRB-1B_L-Meq were significantly higher than those infected with vRB-1B_S-Meq and vRB-1B_Meq (Figure 4A,C). These data suggest that insertion in the transactivation domain does not affect the growth kinetics in the early phase of infection but resulted in a higher viral load during latency and in the transformation phases of infection. However, deletion in the transactivation domain did not influence the replication kinetics of rMDVs. 

### 3.5. Pathogenicity of rMDVs In Vivo

**Figure 5:**
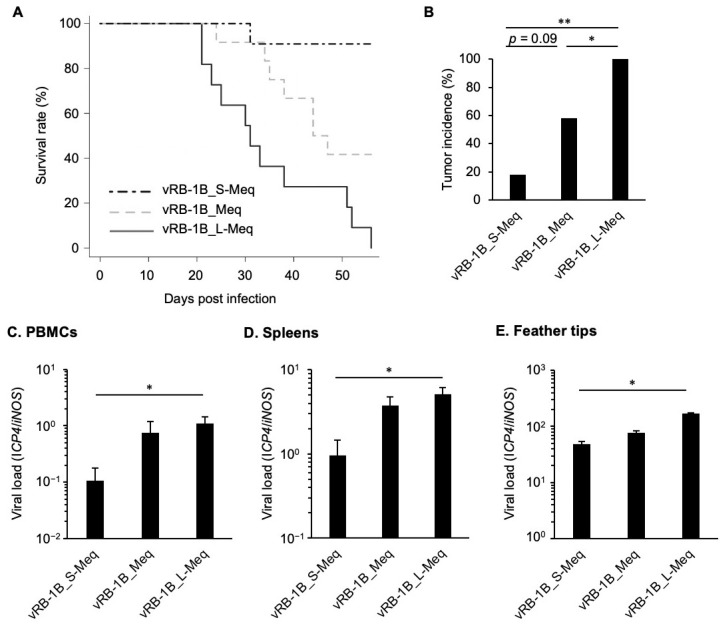
Mortality and tumor incidence in chickens infected with recombinant MDVs. (**A**) Survival rate in chickens infected with recombinant Marek’s disease viruses (rMDVs). Asterisks indicate significant differences. (** *p* < 0.01, * *p* < 0.05; log-rank test). (**B**) Tumor incidence in chickens infected with rMDVs throughout the study period. Asterisks indicate significant differences (** *p* < 0.01, * *p* < 0.05; Fisher’s exact test). The viral loads in (**C**) peripheral blood mononuclear cells, (**D**) spleens, and (**E**) feather tips from chickens infected with the rMDVs were determined by quantitative polymerase chain reaction. Asterisks indicate significant differences (** *p* < 0.01; Kruskal–Wallis test).

To determine whether insertion and deletion in the transactivation domain affect pathogenicity, we monitored the survival rate and tumor incidence in chickens infected with the rMDVs. The vRB-1B_L-Meq was associated with the highest mortality and tumor incidence, whereas the mortality and tumor incidence of vRB-1B_S-Meq were lower than those of vRB-1B_L-Meq and vRB-1B_Meq (Table 3, Figure 5A,B). In addition, we compared viral loads in the PBMCs, spleens, and feather tips collected from chickens infected with each rMDV at the experimental endpoint (euthanized chickens with clinical signs during the experimental period and without clinical signs at 56 dpi). Higher viral loads were observed in the samples from chickens infected with vRB-1B_L-Meq, whereas the viral loads of vRB-1B_S-Meq were significantly lower in all samples (Figure 5C–E). Thus, the pathogenicity of rMDVs was apparently dependent on the length of the transactivation domain.

### 3.6. Pathogenicity of rMDVs in Vaccinated Chickens

**Figure 6:**
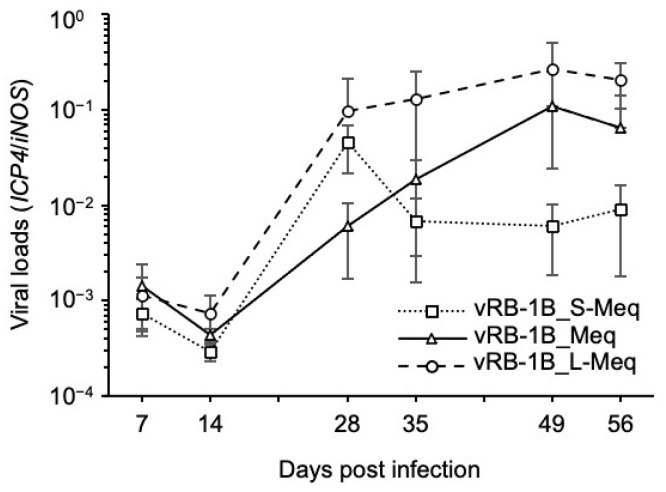
Replication of recombinant MDVs in vaccinated chickens. The chickens were vaccinated and superinfected with vRB-1B_S-Meq, vRB-1B_Meq, or vRB-1B_L-Meq. The viral loads in whole blood were monitored using quantitative polymerase chain reaction with primers specific to the *infected cell protein 4* (*ICP4*) gene of Marek’s disease virus. The growth kinetics among the groups were analyzed using the Kruskal–Wallis test.

Finally, we characterized the pathogenicity of each rMDV in HVT-vaccinated chickens to further characterize the pathogenicity enhanced by the insertion in the transactivation domain. At 28 dpi, no significant difference was observed in the viral loads in whole blood of the vaccinated chickens infected with the vRB-1B_L-Meq strain and other rMDV strains (Figure 6); however, the viral loads in the PBMCs of chickens infected with vRB-1B_L-Meq alone were higher than those of chickens infected with other rMDV strains at 28 dpi (Figure 5A), suggesting that HVT vaccination reduced the viral loads in chickens infected with the vRB-1B_L-Meq strain. However, the viral loads in chickens infected with the vRB-1B_L-Meq strain were higher than those of chickens infected with other rMDVs in the later phase of infection, although the difference in the growth kinetics of rMDVs was not statistically significant (Figure 6). Clinical signs were observed in 2 of the 12 chickens infected with vRB-1B_L-Meq during the experimental period, whereas chickens infected with vRB-1B_Meq or vRB-1B_S-Meq did not exhibit any clinical signs (Table 4). In addition, tumors were observed in 4 of the 12 chickens infected with vRB-1B_L-Meq at termination; however, in the other groups, a tumor was observed in only one chicken infected with vRB-1B_S-Meq (Table 4). These data support the observation that insertion in the transactivation domain of Meq contributes to the increased pathogenicity of this virus.

## 4. Discussion

In this study, we investigated the pathogenicity of rMDVs encoding Meq with insertion or deletion in the transactivation domain of Meq in order to better understand the contribution of Meq to MDV virulence. We compared the transactivation activities among RB-1B-Meq, wild-type S-Meq (Kgw-c2), wild-type L-Meq (CVI988), S-Meq (RB-1B), and L-Meq (RB-1B). S-Meq (RB-1B) and L-Meq (RB-1B) showed higher activities than those of wild-type S-Meq and L-Meq, respectively. These data are consistent with the results that several amino acid substitutions in Meq, such as a glutamic acid-to-lysine substitution, a tyrosine-to-aspartate substitution, and a proline-to-alanine substitution at positions 77, 80, and 217, respectively, contributed to the higher transactivation activity of Meq [21,22] However, the transactivation activities of S-Meq (RB-1B) and L-Meq (RB-1B) were higher than that of the parental RB-1B-Meq. Insertion and deletion into the transactivation-associated domain caused an increase and decrease in the number of PRRs, respectively [19,20]. The PRR, minus the last 39 aa of Meq has been considered to exhibit a transrepressive effect [14], and therefore, an increase or decrease in the number of PRRs was predicted to induce a reduction or increase in the transactivation activity. We previously reported that deletion in the transactivation domain could enhance transactivation activity [20]. However, the L-Meq (RB-1B) exhibited the highest transactivation potential among the Meq constructs, contrary to the PRR theory. According to a previous study, an rMDV encoding CVI-988-L-Meq demonstrated higher pathogenicity than did CVI-988-Meq [30], and these data imply that the insertion in the transactivation domain enhanced the Meq functions related to tumorigenesis. Thus, the enhancement of the transactivation activity observed in L-Meq (RB-1B) seemed to be caused by an unknown mechanism(s) different from the previously suggested theory. As the PRR is theorized to be highly disordered in structure and contains likely binding sites for cellular proteins, alterations in this sequence could have myriad consequences for differential binding of factors. Further analyses are required to clarify the role of PRRs in the protein functions of Meq.

The rMDVs encoding RB-1B-Meq, S-Meq (RB-1B), or L-Meq (RB-1B) exhibited no difference in viral replication in vitro, because Meq was not involved in lytic replication in the infected chickens [18]. In vivo experiments revealed that the viral loads of rRB-1B_L-Meq were higher than those of other rMDVs in the later phases of infection, which could be explained by virus reactivation and/or an increased number of transformed cells upon disease progression. Indeed, vRB-1B_L-Meq caused the highest tumor incidence compared with the other rMDVs. In addition, the increase in viral loads in the feather tips from chickens infected with rRB-1B_L-Meq could facilitate more efficient viral shedding, because the feather follicle epithelium is a site for the production of cell-free viruses [40]. Thus, insertion within the transactivation domain could enhance MDV pathogenicity and efficient virus transmission.

In the present study, vRB-1B_L-Meq showed the highest mortality and tumor incidence rates. Conradie et al. previously reported that an RB-1B-based rMDV encoding wild-type L-Meq from CVI988 was more virulent than an rMDV encoding wild-type Meq from CVI988 [30]. Thus, insertion in the transactivation domain could enhance MDV pathogenicity due to the increase in transactivation activity. In contrast, vRB-1B_S-Meq exhibited the lowest mortality rate and tumor incidence, although the transactivation activity of S-Meq (RB-1B) was higher than that of RB-1B-Meq. Meq has been reported to have several functions, including inhibition of the cGAS–STING pathway [41], interaction with tumor suppressors [42], and transcriptional regulation [43,44], and deletion in the transactivation domain may reduce some of these protein functions or other unknown functions. For instance, Meq could inhibit the cGAS-STING DNA-sensing pathway, which plays a vital role in innate immunity in chickens [41] by interacting with STING through the C-terminal domain in Meq, thereby suppressing the expression of type 1 interferon [41]. Therefore, insertion and deletion in the transactivation domain in Meq may affect the immunosuppressive effects through the interaction of STING with the C-terminal domain of Meq. Further analyses are required to elucidate the molecular mechanisms by which insertion and deletion in the transactivation domain of Meq modulate MDV pathogenicity.

In the present study, we found that insertion in the transactivation domain causes enhanced pathogenicity, whereas deletion results in reduced pathogenicity. Indeed, virulent MDV strains circulating in Australia encode an L-Meq and exhibit the features of MDV strains with high virulence [24]. Therefore, insertion in the transactivation domain may be responsible for the high virulence of MDV strains in Australia, in addition to the polymorphisms in Meq. However, there is a discrepancy between the emergence of MDV strains with enhanced virulence and the years in which the MDV strains with insertions and deletions were detected when we considered the timeline of MDV evolution. MDV strains encoding L-Meq were originally found in field strains from the 1960s and 1970s with low virulence in the US and an attenuated vaccine strain, CVI988 [19], whereas MDV strains with S-Meq have been reported in the field since the 2010s. Moreover, a very short isoform of Meq, which encodes one copy of PRR in the transactivation domain, was reported in a virulent MDV strain circulating in Iran [45]. Although the pathogenicity of such field strains having the S-Meq is uncertain, MDV strains with short isoforms of Meq have emerged more recently than MDV strains with L-Meq and Meq along the timeline. 

In the present study, however, MDV pathogenicity was reduced by deletion in the transactivation domain, which was inconsistent with the tendency of MDV strains to exhibit enhanced virulence in the field. The reduced virulence following the loss of sequences in Meq may result in the prolonged survival of chickens infected with MDV, thereby leading to the efficient virus shedding and maintenance of MDV in the poultry houses; it may indicate an aspect of virus evolution aimed at symbiosis with the host. In addition, although the polymorphisms and insertion/deletion in Meq could be factors contributing to MDV virulence, other viral factors may be involved in the evolution of MDV. This study could improve our understanding of the mechanisms by which MDV alters the virulence and survival strategy of MDV in the environment.

As limitations in the present study, we could not identify why the deletion in the transactivation domain causes the reduced pathogenicity of Meq, despite the enhanced transactivation activity. On the other hand, the insertion in the transactivation domain enhanced the transactivation activity, contrary to the previously suggested theory. Thus, the PRR domain in Meq appears to have unknown functions separate from a role in transcriptional regulation. Therefore, to better understand the molecular basis of MDV pathogenicity and its enhanced virulence, it is necessary to further investigate the functions of Meq and the proteins that could interact with the various Meq isoforms. In addition, in the present study, we used the rMDVs encoding S-Meq that the amino acid mutations introduced, and we did not evaluate the pathogenicity of rMDV encoding wild-type Kgw-c2 S-Meq by comparing with that of rMDV encoding S-Meq (RB-1B). Therefore, to evaluate the risks of future outbreaks caused by MDV strains encoding S-Meq, the pathogenicity of field strains encoding S-Meq with some amino acid substitutions toward higher virulence should be investigated. Likewise, to investigate the contribution of amino acid substitutions at positions other than the insertion in the transactivation domain to the MDV pathogenicity, it is necessary to compare the pathogenicity of rMDV encoding wild-type L-Meq with that of rMDV encoding L-Meq (RB). Additionally, we used the RB-1B-based rMDVs to determine whether the insertion or deletion contribute to the pathogenicity, and then, the mutations were introduced to L-*meq* and S-*meq* genes to match the sequences with that of the *meq* gene of RB-1B. Therefore, the possibility that the artificially introduced mutations affected the viral biology should be considered. The use of rMDVs from other strains, such as the CVI988-based rMDVs, should be tested to determine their effect on pathogenicity.

## Figures and Tables

**Table 1:** Introduction of the mutation into the S-*meq* and/or L-*meq* genes.

Position in Meq	Substitution	Primers	
71	serine–to–alanine	F	5′-GAA TCG TGA CGC CGC TCG GAG AAG ACG-3′
		R	5′-CGT CTT CTC CGA GCG GCG TCA CGA TTC-3′
77, 80	glutamic-acid–to–lysine, tyrosine–to–aspartic-acid	F	5′-CGG AGA AGA CGC AGG AAG CAG ACG GAC TAT GTA GAC AAA C-3′
		R	5′-GTT TGT CTA CAT AGT CCG TCT GCT TCC TGC GTC TTC TCC G-3′
114, 115	cysteine–to–arginine, alanine–to–valine	F	5′-GAG TGC ACG TCC CTG CGT GTA CAG TTG GCT TGT CA-3′
		R	5′-TGA CAA GCC AAC TGT ACA CGC AGG GAC GTG CAC TC-3′
217	alanine–to–proline	F	5′-ATC TGT ACC CCC CCT CCT CCC GAT G-3′
		R	5′-CAT CGG GAG GAG GGG GGG TAC AGA T-3′
326	isoleucine–to–threonine	F	5′-GTT TCC CTC GGA TAC TCA GTC TAC GGT CT-3′
		R	5′-AGA CCG TAG ACT GAG TAT CCG AGG GAA AC-3′

**Table 2:** Primers used for reverse transcription-polymerase chain reaction and quantitative polymerase chain reaction analyses.

Gene	Sequence	Application
*meq*	F 5′-AGT TGG CTT GTC ATG AGC CAG-3′	RT-PCR
	R 5′-TGT TCG GGA TCC TCG GTA AGA-3′	
*ICP4*	F 5′-GCA TCG ACA AGC ACT TAC GG-3′	qPCR
	R 5′-CGA GAG CGT CGT ATT GTT TGG-3′	
*iNOS*	F 5′-GAG TGG TTT AAG GAG TTG GAT CTG A-3′	qPCR
	R 5′-TTC CAG ACC TCC CAC CTC AA-3′	

Abbreviations: qPCR, quantitative polymerase chain reaction; RT-PCR, reverse transcription polymerase chain reaction.

**Table 3:** Survival rate and tumor incidence at 56 days post-infection.

rMDV	Survival Rate	Tumor Incidence
vRB-1B_S-Meq	90.9% (10/11)	18.2% (2/11)
vRB-1B_Meq	41.7% (5/12)	58.3% (7/12)
vRB-1B_L-Meq	0% (11/11)	100% (11/11)

**Table 4:** Survival rate and tumor incidence in vaccinated chickens at 56 days post-infection.

rMDV	Survival Rate	Tumor Incidence
vRB-1B_S-Meq	100% (8/8)	12.5% (1/8)
vRB-1B_Meq	100% (9/9)	0% (0/9)
vRB-1B_L-Meq	83.3% (10/12)	33.3% (4/12)

## Data Availability

Data are contained within the article.

## Supplementary Materials

The following supporting information can be downloaded at: https://www.mdpi.com/article/10.3390/v14020382/s1, Figure S1: The original uncropped image related to Figure 2B; Figure S2: The original uncropped image related to Figure 3A.
