# Supplementary material for: Effect of Insertion and Deletion in the Meq Protein Encoded by Highly Oncogenic Marek’s Disease Virus on Transactivation Activity and Virulence
Source: Viruses. 2022 Feb 14;14(2):382. doi: 10.3390/v14020382 (PMC8876991; doi:10.3390/v14020382)
Supplement: Supplementary file 1 [file viruses-14-00382-s001.zip › viruses-1591992-supplementary.pdf]

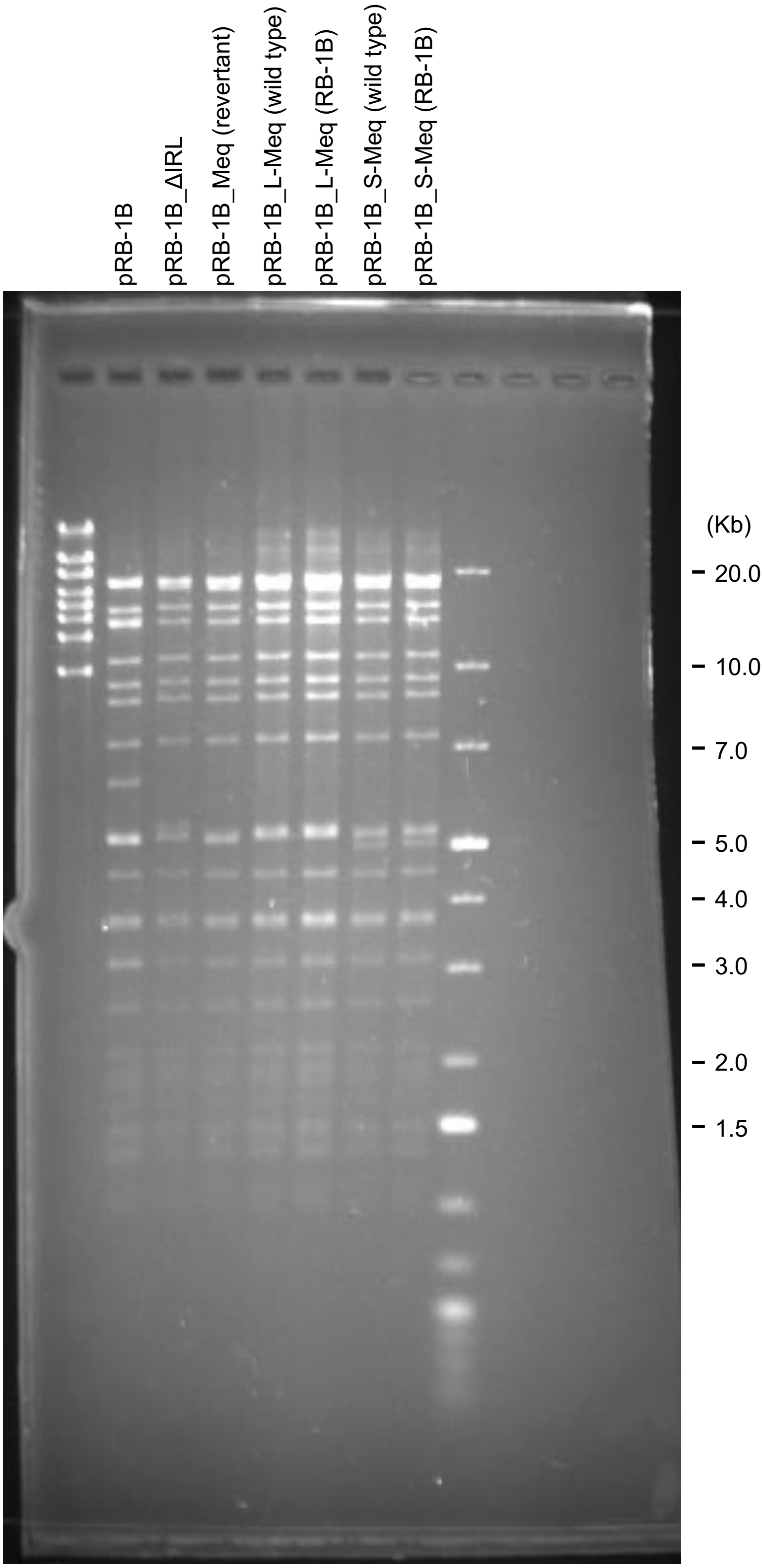


**Supplementary Figure S1.** The original uncropped image related to Figure 2B.

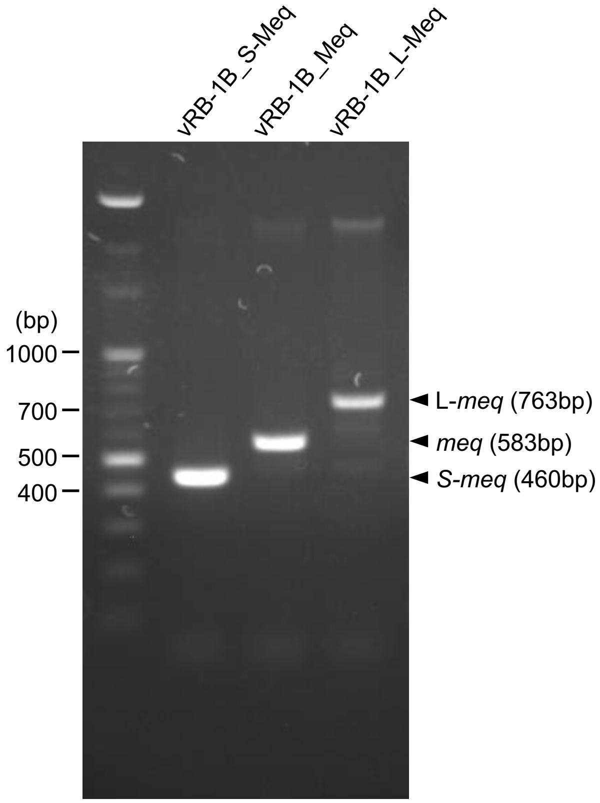


**Supplementary Figure S2.** The original uncropped image related to Figure 3A.
